# Frontloading of stress response genes enhances robustness to environmental change in chimeric corals

**DOI:** 10.1186/s12915-022-01371-7

**Published:** 2022-07-26

**Authors:** Jeremie Vidal-Dupiol, Erwan Harscouet, Dor Shefy, Eve Toulza, Olivier Rey, Jean-François Allienne, Guillaume Mitta, Baruch Rinkevich

**Affiliations:** 1 IHPE, Univ Montpellier, CNRS, IFREMER, Univ Perpignan Via Domitia, Montpellier, France; 2 Department of Life Sciences, Ben-Gurion University, Eilat Campus, 84105 Be’er Sheva, Israel; 3 Israel Oceanography & Limnological Research, National Institute of Oceanography, Tel Shikmona, PO Box 9753, 3109701 Haifa, Israel; 4 The Interuniversity Institute of Eilat, P.O.B 469, 88103 Eilat, Israel; 5 IHPE, Univ Montpellier, CNRS, IFREMER, Univ Perpignan Via Domitia, Perpignan, France; 6 Univ Polynesie Francaise, ILM, IRD, Ifremer, Tahiti, F-98719 French Polynesia, France

**Keywords:** Chimera, Corals, Global change, Transcriptomics, Plasticity/robustness, Environmental change

## Abstract

**Background:**

Chimeras are genetically mixed entities resulting from the fusion of two or more conspecifics. This phenomenon is widely distributed in nature and documented in a variety of animal and plant phyla. In corals, chimerism initiates at early ontogenic states (larvae to young spat) and results from the fusion between two or more closely settled conspecifics. When compared to genetically homogenous colonies (non-chimeras), the literature has listed ecological and evolutionary benefits for traits at the chimeric state, further positioning coral chimerism as an evolutionary rescue instrument. However, the molecular mechanisms underlying this suggestion remain unknown.

**Results:**

To address this question, we developed field monitoring and multi-omics approaches to compare the responses of chimeric and non-chimeric colonies acclimated for 1 year at 10-m depth or exposed to a stressful environmental change (translocation from 10- to 2-m depth for 48h). We showed that chimerism in the stony coral *Stylophora pistillata* is associated with higher survival over a 1-year period. Transcriptomic analyses showed that chimeras lose transcriptomic plasticity and constitutively express at higher level (frontload) genes responsive to stress. This frontloading may prepare the colony to face at any time environmental stresses which explain its higher robustness.

**Conclusions:**

These results show that chimeras are environmentally robust entities with an enhanced ability to cope with environmental stress. Results further document the potential usefulness of chimeras as a novel reef restoration tool to enhance coral adaptability to environmental change, and confirm that coral chimerism can be an evolutionary rescue instrument.

**Supplementary Information:**

The online version contains supplementary material available at 10.1186/s12915-022-01371-7.

## Background

**Fig. 1:**
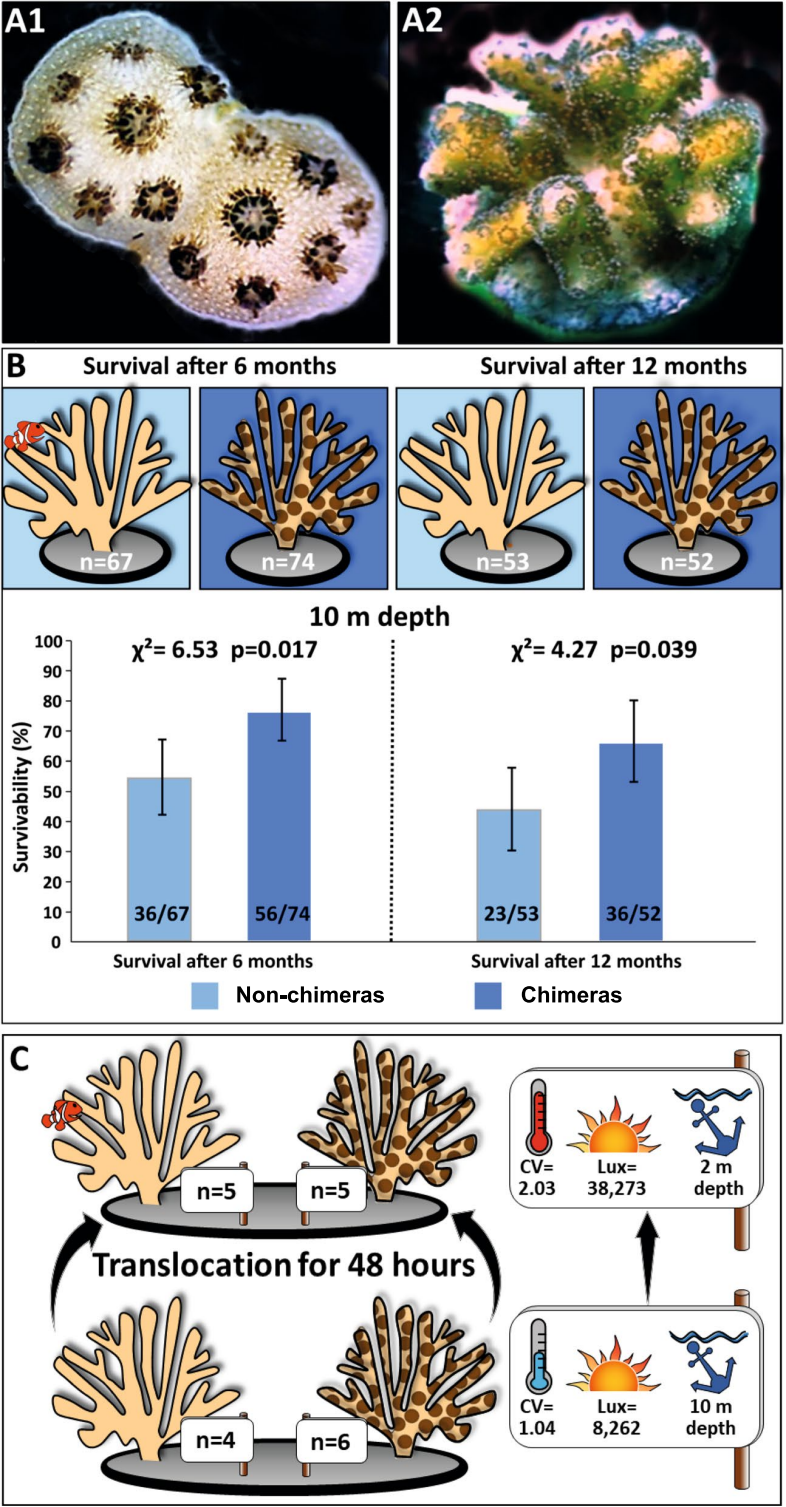
Chimeras display higher survival *in natura*. (**A**) *S. pistillata* chimeras (A1) following fusion between two genetically different spats that are still distinguishable; (A2) a 1-year-old juvenile chimera resulting from the fusion of two genetically different spats. Both partners are intermingled and form a single colony with the same morphology as non-chimeras. (**B**) Survival of non-chimeras (unicolor coral) and chimeras (bi-color coral) over 6 and 12 months of exposure to regular and natural environmental conditions at 10-m depth assessed at the 95% confidence intervals using the “Cloper – Pearson” method and chi-square. (**C**) Translocation experiment, from 10 to 2m, used to induce an abrupt environmental change characterized by higher temperature variation (CV. coefficient of variation) and average light intensity (μ in lux)

Coral chimeras are defined as genetically mixed entities formed by the fusion of two or more conspecifics [1, 2] (Fig. 1A). Although coral chimerism was discovered more than a century ago [3], it has been poorly studied. Interestingly, several studies revealed (i) the presence of adult coral chimeras in the field [4–7] and (ii) that the fusion process leading to coral chimerism is limited to planulae and young spat life stages [8–12] (Fig. 1A1).

Ecological benefits, including increased survival and growth rates, have been associated with the chimeric status [3, 5, 6, 8, 13]. In addition, asexually developing chimeric larvae [6] and chimerism at the planktonic life stage (fusion between allogeneic planulae [12, 13]) may enhance fitness through the maintenance of genetic diversity in populations [6]. A chimeric entity may also possess a wider range of phenotypical expression [1, 2, 14] driven by higher genetic and non-genetic diversity [15, 16]. This higher level of diversity may maximize the resilience of the coral chimera resulting in a higher potential to withstand environmental changes. These theoretical considerations suggest coral chimera as an important, yet still neglected, evolutionary rescue instrument [3].

At the transcriptome level, the wider range of phenotypical expression can take different forms and several patterns of transcriptomic plasticity were already associated with stress tolerance [17]. Frontloading is defined as a higher baseline expression but reduced plasticity of some genes including those associated with stress responses (e.g., HSP70, TRAF, ROX detoxifier) [18]. Frontloading was first identified in corals living in fluctuating and stressful thermal conditions and displaying reduced bleaching sensitivity [18]. Later, frontloading was reported in corals regularly exposed to high temperatures [19] or to extreme annual thermal variation and maxima [20]. Dampening is defined as reduced expression plasticity. Dampening of metabolic and ribosomal processing genes during the stress response was associated to coral thermal tolerance [21]. The combination of different patterns may also be recorded. In corals from Oman, a mix between frontloading and higher plasticity (genes with higher basal expression levels and higher plasticity) was observed and associated with a strong thermal tolerance.

To first test if coral chimeras display a higher stress tolerance, we produced experimentally chimeric and non-chimeric corals and acclimated them for 1 year at 10-m depth *in natura*. Next, to understand the molecular mechanisms underlying the higher survival measured for chimeras, we exposed both chimeric and non-chimeric corals to a rapid environmental change (translocation from 10- to 2-m depth) and analyzed them using a multi-omics approach. This analysis revealed high and constitutive expression of stress response genes (frontloading [18]) in chimeras, while non-chimeric corals displayed a classical induction of stress response genes. This phenomenon can explain the robustness of chimeras, as chimeras are constitutively prepared to withstand environmental stress.

## Results

### Chimeras display higher survival in natura

To investigate potential fitness differences between chimeric and non-chimeric *Stylophora pistillata* colonies, we assessed survival rates of experimentally produced chimeras and non-chimeras acclimated *in natura* at a depth of 10 m. Survival rates at 10-m depth were higher in chimeric colonies after both 6 and 12 months (Fig. 1B; *χ*^2^ = 6.53, *p* < 0.05; χ^2^ = 4.27, *p* value < 0.05, respectively), indicating that chimeras have a fitness advantage.

To investigate whether the higher survival of chimeras is due to better resistance to environmental stress, we translocated nine chimeras (from which five were validated by microsatellite, see below) and five non-chimeras from 10- to 2-m depth for 48 h, exposing them to sudden and higher temperature and light fluctuations (Fig. 1C; Additional file 1, Tables S1 and S2). At 2-m depth, the temperature ranged between 25.03 and 26.78°C while the temperature at 10-m depth ranged between 24.84 and 25.90°C. Differences between the two depths were even stronger for light intensities with an average sun radiation fourfold higher at 2 m (38,273 lux) than at 10 m (8262 lux). During diurnal light peak (10–16h), the maximum illumination level was sevenfold higher at 2m (85,422 lux) than at 10 m (12,400 lux). No coloration changes (e.g., bleaching or darkening) were observed for any of the corals during the experiment (coral reef watch color chart) which confirm that strong differences in Symbiodiniacaea density would not bias gene expression analyses.

Metabarcoding and dual RNA-seq were performed to check whether the above stressful environmental changes or chimeric status fostered a change in the coral colony microbiome or in the holobiont transcriptome. Before addressing this question, we validated the chimeric/non-chimeric status of the 27 colonies used for the translocation experiments using previously developed microsatellites (Additional file 1: Table S3). Among the 18 chimeras used, 11 were validated and displayed at least one locus with three or more alleles showing at least two co-occurring genomes in one colony. All seemingly non-chimeras (displaying just mono- or biallelic loci) but that could still present the undetectable state of microchimerism were removed from the metabarcoding and dual RNA-seq analyses. At the end, six and five validated chimeras and four and five non-chimeras were exposed to the 10-m or 2-m depth treatments, respectively (Fig. 1C). This higher allelic number per locus in chimeras was further confirmed using multiple nucleotide polymorphisms (MNPs) derived from the RNA-seq data (see below). This last approach showed that chimeras presented a significantly higher relative quantity of multiallelic MNPs (*n* allele > 3) than non-chimeric colonies (Mann–Whitney *U* test; *U* = 18, *p* value < 0.05; Additional file 1: Table S3).

### No effect of chimerism or translocation on the microbial community

To check whether such stressful environmental change or chimeric status fostered a potential change in symbiont communities, we performed ITS2 amplicon sequencing for chimeras and non-chimeras exposed at 10- and 2-m depth. After clustering and filtering for operational taxonomic units (OTUs) containing less than 1% of sequence tags, a single ITS2 OTU, representing 97.7% of the dataset, was revealed (Additional file 1: Table S4). Taxonomic affiliation using blast comparisons to the NCBI nr/nt database enabled the identification of *Symbiodinium* sp. (former *Symbiodinium microadriaticum* clade A1 *S. pistillata* isolate Eilat; GenBank MH211592.1) with 100% identity over the whole amplicon sequence. Thus, we found no major differences between the symbiont communities hosted in chimeric or non-chimeric corals at both depths.

We then investigated bacterial microbiome diversity using 16S metabarcoding. We obtained 2,073,020 informative clusters (range: 28,000–263,000 per sample) from the 20 constructed libraries. After clustering and singleton filtering, 8016 OTUs were subjected to affiliation by blast against the SILVA SSU database [22] (Additional file 1: Table S5). Alpha diversity (species richness, Chao and Shannon) and beta diversity (Bray–Curtis dissimilarity) were calculated and plotted for chimeras vs. non-chimeras, with and without depth effect (Additional file 2: Fig. S1A and B). The sole significant difference observed was a lower Shannon diversity index for the non-chimeric corals at 2-m depth (MANOVA *p* = 0.00758; Additional file 2: Fig. S1A3). This difference was mainly driven by a single sample displaying low diversity compared to the other samples. The mean alpha diversity was higher in chimeras than in non-chimeric corals at both depths, but the difference was non-significant. Regarding the beta diversity, neither the depth nor the chimeric status had a significant effect (Bray–Curtis dissimilarity index; Additional file 2: Fig. S1B). Taxonomic composition at the family level was consistent in all the conditions and confirmed the lack of association between bacterial community composition and chimeric status or depth (Additional file 2: Fig. S1C).

Together, these data show that chimeric *S. pistillata* colonies have higher survival than non-chimeric counterparts and that this difference is not likely due to differences in symbiont communities or bacterial microbiome.

### Chimerism induces transcriptomic changes in *S. pistillata* and *Symbiodinium* sp.

We next investigated potential changes in gene transcription in chimeras and non-chimeras at the two different depths at the whole transcriptome scale using a RNA-seq approach. This yielded an average of 25.25 ± 0.8 million paired-end reads per sample, of which ~1.2% raw sequences were discarded after the preprocessing steps (trimming, quality filtering, and adaptor removal). Most (68 ± 4.0%) of these filtered reads were uniquely mapped and properly paired on the reference genome of *Stylophora pistillata* [23] or *Symbiodinium microadriaticum* [24]. No significant differences between samples in the proportion of reads mapped to *S. microadriaticum* were observed which confirms the absence of bleaching (10m vs. 2m Mann–Whitney *U* test, *U* = 47.5, *p* value = 0.88; chimeras vs. non-chimera, *U* = 44.5, *p* value = 0.73; Additional file 1: Table S6). Hierarchical clustering analysis performed on the distance matrix based on the whole transcriptome of each samples controlled for the mother colony of origin showed that for both, *S. pistillata* and *S. microadriaticum*, samples clustered first in function of their chimeric status and then by the treatment (Additional file 2: Fig. S2A & B). This last result is confirmed by principal component analysis (PCA) showing that the first axis mostly explains the chimeric status while the second axis explains the depth (Additional file 2: Fig. S3A & B). These first results showed the strong transcriptomic effects associated to the chimeric status and then to the translocation.

**Fig. 2:**
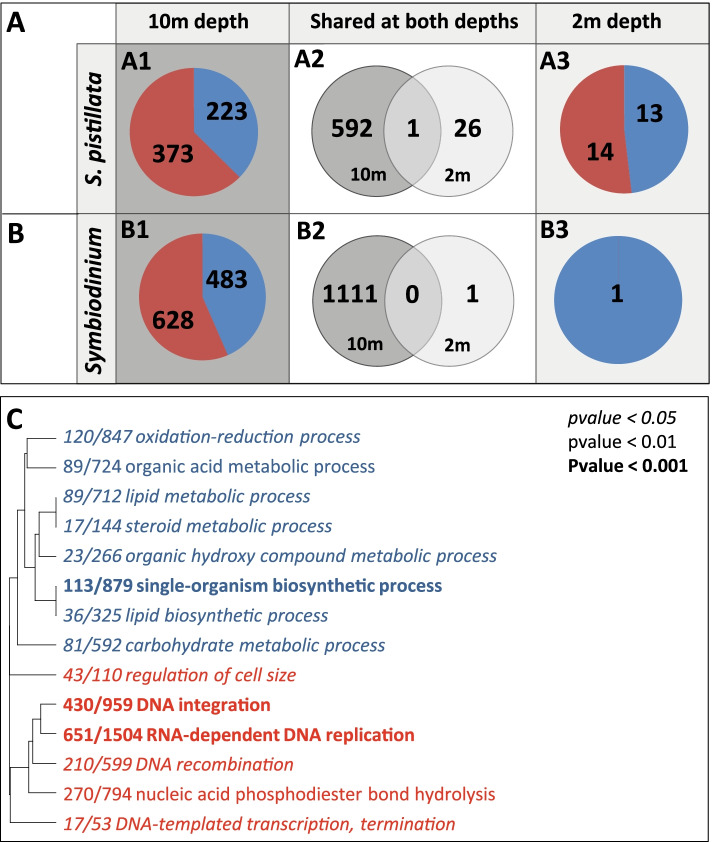
Chimerism induces transcriptomic changes in *S. pistillata* and *Symbiodinium* sp. (**A**, **B**) Number of genes differentially expressed, between non-chimeras and chimeras (**A1**–**A3**) or between *Symbiodinium* sp. hosted in non-chimeras or chimeras (**B1**–**B3**): (**A1**, **B1**) When acclimated for 1 year at 10-m depth; (**A3**, **B3**) after 48h of exposure to a sudden and stressful environmental change (translocation to 2-m depth) and (**A2**, **B2**) shared between the two environmental treatments. (**C**) Biological process (GO terms) significantly enriched (Mann–Whitney *U* test) in *S. pistillata* at 10m. Over-expressed/represented genes/GO terms are in red and under-expressed/represented genes/GO terms in blue (italic: *p* value < 0.05; normal: *p* value < 0.01; bold: *p* value < 0.001). *x*/*y* reflects the number of genes with a |log2FC| > 2 in the GO category/the total number of genes in the GO category

When acclimated for 1 year at 10-m depth, chimeras vs. non-chimeras differentially expressed 596 genes (DEGs; FDR < 0.05; 373 over-expressed; 223 under-expressed in chimeras; Fig. 2A1; Additional file 1: Table S7). When exposed to a sudden environmental stress, 48h at 2 m, only 27 genes were differentially expressed (14 over-expressed; 13 under-expressed; Fig. 2A3). Only one gene was differentially expressed between chimeras and non-chimeras at both 10-m and 2-m depth (Fig. 2A2).

Gene ontology (GO) term enrichment analysis (GO_MWU; *p* value < 0.05) that compared the transcriptomes of the acclimated chimeras and non-chimeras at 10-m depth has revealed that 14 biological process were significantly enriched with 6 GO terms over-represented among genes over-expressed and 8 among genes under-expressed (Fig. 2C). The over-represented GO terms highlighted that chimeras are characterized by a higher level of processes associated to DNA and RNA replication, recombination, and integration, suggesting a high activity of transposable elements. The GO terms over-represented among genes under-expressed (Fig. 2C) showed a lower metabolic activity in chimera (GO terms: “organic acidmetabolic process,” “lipid metabolic process,” “carbohydrate metabolic process,” etc.). Individual screening of each DEGs confirmed the trends revealed at the biological process level, with chimeras displaying over-expressions of eight transposable elements (TEs) with log2 fold change (log2FD) between 9.53–2.2 (Additional file 1: Table S7). Surprisingly, it also revealed that approximately 9% of the DEGs were genes encoding proteins involved in the extracellular matrix, cell proliferation, wound healing, and tissue remodeling (Additional file 1: Table S7). These genes displayed a stochastic expression pattern composed by a mix of over- and under-expressed genes. Activation of immune pathway was also identified with the over-expression in chimera of eight different TNF receptor-associated factors (TRAF), myeloid differentiation primary response 88 (MYD88), stimulator of interferon gene (SIG), and interferon regulatory factor (IRF). Inflammation was interestingly characterized by the under-expression of two tyrosinase (melanization pathway) as well as the under-expression of the complement factors C3 and C1q (Suppl. Table 7).

GO term enrichment analysis using the GO_MWU script revealed that in chimeras exposed at 2 m, no GO terms were significantly enriched. Among the 27 DEGs (Additional file 1: Table S8), a gene encoding an acid ceramidase involved in the sphingolipid biosynthesis pathway (a regulatory pathway involved in many cellular processes including innate immunity, acute inflammatory responses, and activation of immune cells and apoptosis [25]) and two genes encoding proteins of the extracellular matrix were under-expressed.

The stochastic expression patterns of genes involved in cell–cell interactions and tissue organization probably highlight physical interactions between the genotypes co-occurring within the chimera. The over-expression of TEs in chimeras suggests that these physical interactions between cells with different genetic backgrounds may lead to a stress at the genome scale. Therefore, chimerism appears to induce transcriptomic changes characteristic of internal conflict that may occur between the different co-occurring genotypes.

When acclimated for 1 year at 10-m depth, differential gene expression analysis showed that 1111 genes were differentially expressed (628 over-expressed; 483 under-expressed; FDR < 0.05; Fig. 2B1, Additional file 1: Table S9) in symbiotic algae hosted in chimeras vs. those hosted in non-chimeras. By contrast, only a single gene was over-expressed in chimeras after 48 h at 2 m (Fig. 2B3, Additional file 1: Table S10). No genes were differentially expressed both at 10 and 2 m (Fig. 2B2).

GO_MWU analysis showed that no biological processes were significantly enriched at 10-m or 2-m depth. At 10 m, many DEGs were involved in bicarbonate transport or conversion (e.g., electrogenic sodium bicarbonate cotransporter, carbonic anhydrase), nitrate transport (e.g., high-affinity nitrate transporter), or ion transport (e.g., sodium channel protein type 8, potassium voltage-gated channel protein etc.). This trend suggests an effect of chimerism on the organic/inorganic matter uptake/excretion function of the symbiont (Additional file 1: Table S9).

Gene expression of the *Symbiodinium* sp. is surprisingly affected by the coral chimerism while generally acknowledged as relatively stable. The regulation of biological functions linked to ion transport and nutrient uptake may highlight a change in the trophic interactions between the cnidarian host and its symbiont.

### Chimera frontload stress-responsive genes

**Fig. 3:**
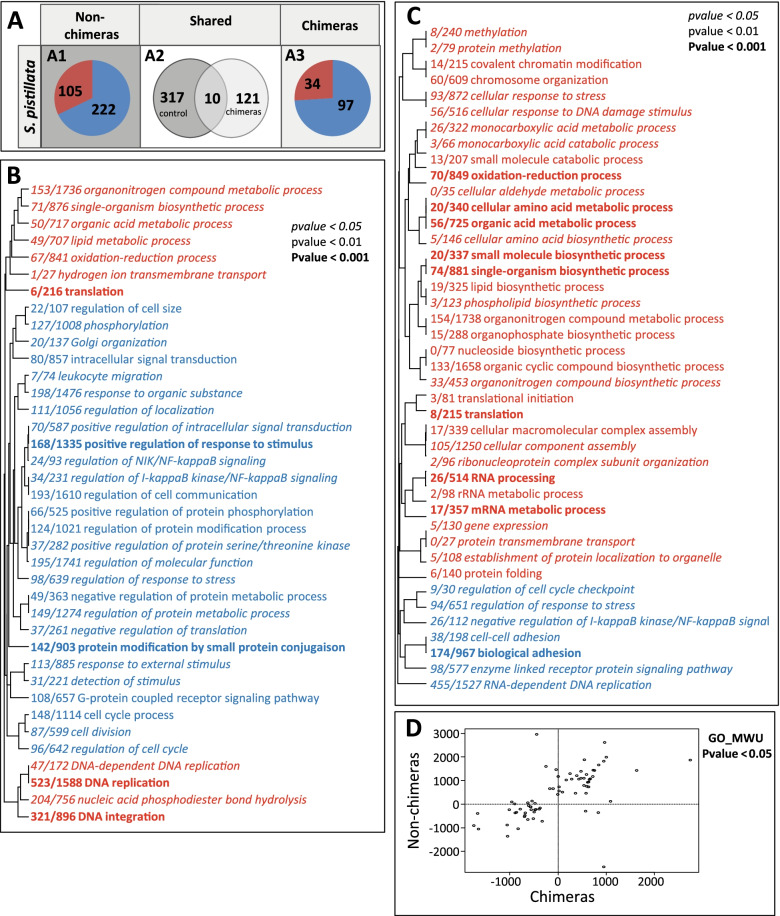
Transcriptomic modifications induced by the translocation in chimeras and non-chimeras of *S. pistillata.* (**A**) Number of genes differentially expressed in response to the translocation in non-chimeras of *S. pistillata* (**A1**) and in chimeras of *S. pistillata* (**A3**) or shared between entities (**A2**). (**B**, **C**) Biological processes (GO terms) significantly enriched (Mann–Whitney *U* test): (**B**) in non-chimeras of *S. pistillata*; (**C**) in chimeras of *S. pistillata*. Over-expressed/represented genes/GO terms are in red and under-expressed/represented genes/GO terms are in blue. *p* values under the 0.05, 0.01, and 0.001 thresholds are indicated in italic, in normal font, and in bold, respectively. *x*/*y* reflects the number of genes with a |log2FC| > 2 in the GO category/the total number of genes in the GO category. (**D**) Delta-rank comparison between the responses of non-chimeras and chimeras

In response to the translocation from 10- to 2-m depth, non-chimeric colonies differentially expressed 327 genes (105 over-expressed and 222 under-expressed; Fig. 3A1; Additional file 1: Table S11). By contrast, chimeras differentially expressed 131 genes (34 over-expressed, 97 under-expressed; Fig. 3A3; Additional file 1: Table S12). Comparisons of these two responses showed that only 10 genes were differentially expressed in both responses (Fig. 3A2).

Enrichment analyses revealed that 38 GO biological processes were significantly enriched (*p* value < 0.05; 11 were over-represented among genes over-expressed and 27 among genes under-expressed) in non-chimeric colonies (Fig. 3B). In chimeras, 42 GO categories were enriched (35 over-represented, 7 under-represented; Fig. 3C). Comparisons between each enrichment results revealed that the overall pattern of responses was close for both entities (Fig. 3D).

In non-chimeras, we identified biological processes classically enriched in coral response to light and temperature stress, including processes associated to reactive oxygen species (ROS) detoxification (e.g., oxidation-reduction process; over-represented), immunity (e.g., regulation of I-kappa B kinase/NF-kappa B signaling, under-represented), and stress response (e.g., regulation of response to stress, under-represented). In chimeras, GO terms such as cellular response to stress and protein folding (over-represented) were also found. At the single gene level, a gene-by-gene screening for stress-responsive genes among the DEGs expressed during the response of both entities revealed more significant over-expressions in non-chimeras of (i) three members of the *HSP* family (e.g., HSP70 and HSF) in the non-chimeras; (ii) 10 (e.g., thioredoxin and peroxiredoxin) and three (e.g., cytochrome P450) ROS scavengers in non-chimeras and chimeras, respectively; (iii) 10 immune-related genes in non-chimeras (e.g., complement C3 and TRAF3); and (iv) four genes involved in cell death pathways (e.g., p53) in non-chimeras (Suppl. Tables 11 and 12). This screening showed that the stress response induced by the translocation in non-chimeras is stronger than in chimeras.

**Fig. 4:**
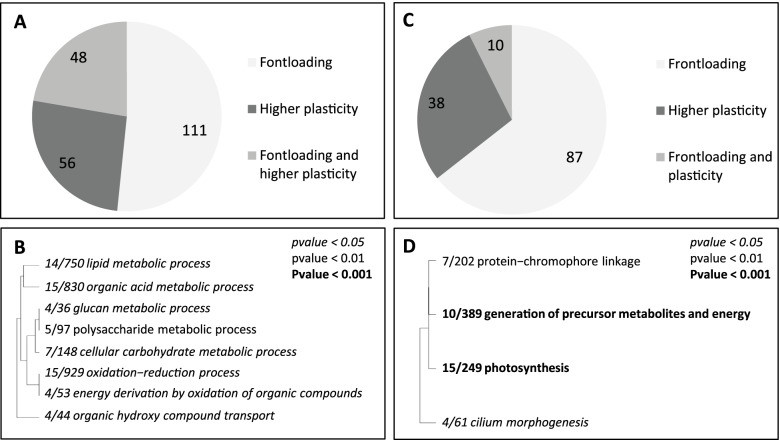
Chimeras frontload stress-responsive genes (**A**, **C**). Expression pattern differences between non-chimeras and chimeras for the genes responding to the translocation in the non-chimeras: **A** for the host and **C** for the symbiont. **B**, **D** GO categories from the biological process roots that are significantly enriched (Fisher exact test; FDR < 0.05), in the frontloaded gene set for the host (**B**) and the symbiont (**D**). *p* values under the 0.05, 0.01, and 0.001 thresholds are indicated in italic, in normal font, and in bold, respectively; *x*/*y* reflects the number of DEGs in the GO category/the total number of genes in the GO category

To better understand the differences in gene expression patterns between non-chimeric colonies and chimeras, we focused on the 327 genes responding to the translocation of the non-chimeric colonies and looked how these genes were expressed in the chimeras acclimated to the environment and in response to the translocation to 2-m depth (Fig. 4A). These differences were highlighted accordingly to their transcriptomic plasticity and using the following categorization: (i) frontloaded genes (i.e., higher basal expression level in chimera), (ii) higher plasticity (i.e., genes with identical or lower basal expression but over- or under-expressed higher in chimeras in response to the translocation), and (iii) frontloaded and higher plasticity (higher basal expression and higher over- or under-expression in chimeras in response to the translocation). The analyses revealed that 215 genes followed one of these patterns: 111 were frontloaded, 56 displayed higher transcriptomic plasticity, and 48 were frontloaded and displayed higher transcriptomic plasticity (Fig. 4A). GO term enrichment analyses were then performed on these different sets of genes.

Significant enrichments were obtained for the frontloaded set only (*p* value < 0.05; Fig. 4B). In particular, 8 GO terms mostly illustrating a higher activity linked to the energetic metabolisms at the basal expression level (e.g., glucan metabolic process, energy derivation by oxidation of organic compounds, etc.) were enriched. Interestingly the GO term oxidation-reduction process was also composed by genes involved in ROS detoxification, a key coral stress response.

At the single gene level, a screening among all the genes displaying a putative adaptive expression pattern in chimera revealed that 5 ROS detoxifiers (e.g., peroxidasin, peroxiredoxin, etc.) were frontloaded and one (a thioredoxin) displayed a higher plasticity. Among the *HSP* family, the HSF and HSP16 genes were frontloaded and the HSP70 gene was frontloaded and higher expressed in response to the translocation. In addition, seven genes involved in apoptosis and cell death pathways (e.g., FAS-associated factor 1 and p53) and 10 immune-related genes (e.g., complement *C3* and galectin) were also frontloaded.

In non-chimeric colonies, the stress response was more potent, but most of the essential genes needed to display an efficient stress response were already expressed constitutively at higher levels in acclimated chimeras and/or were regulated higher. These different forms of transcriptomic plasticity may increase the robustness of chimeras.

### *Symbiodinium* sp. hosted in chimeras and non-chimeras respond differently

**Fig. 5:**
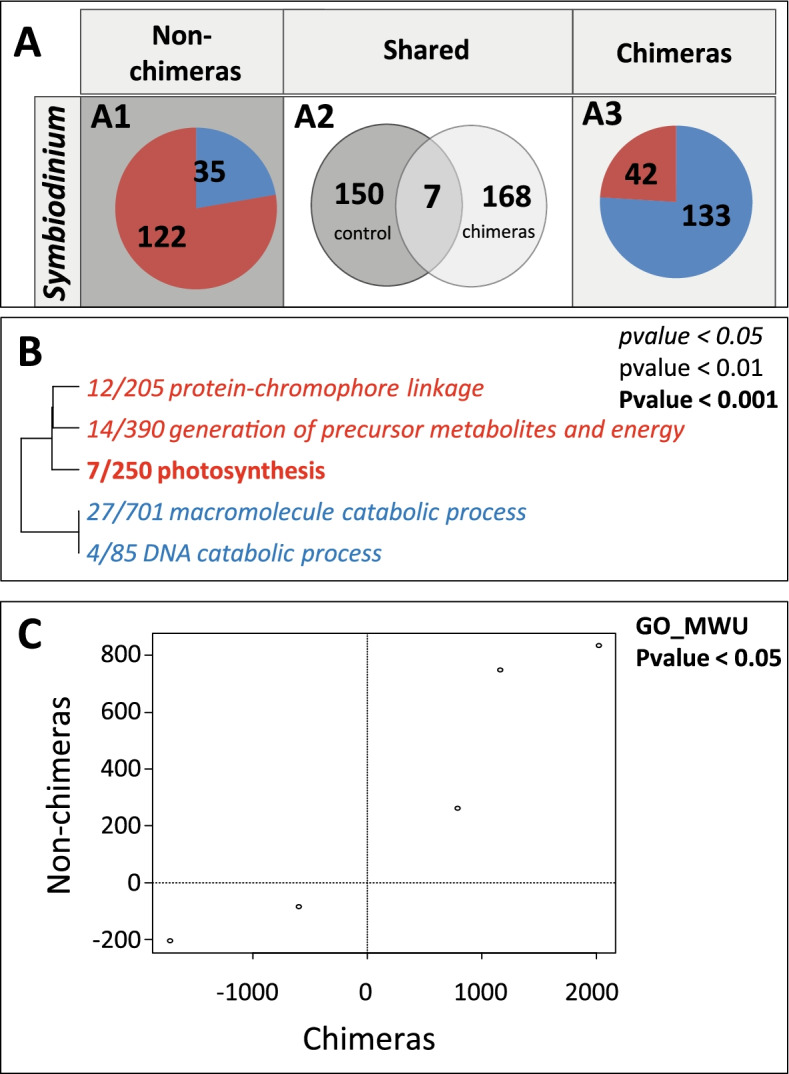
Transcriptomic modifications induced by the translocation in *Symbiodinium* sp. hosted in chimeras and non-chimeras. (**A**) Number of genes differentially expressed in response to the translocation in non-chimeras (**A1**) and in chimeras (**A3**) or shared between entities (**A2**). (**B**) Biological processes (GO terms) significantly enriched (Mann–Whitney *U* test) in non-chimeras. Over-expressed/represented genes/GO terms are in red and under-expressed/represented genes/GO terms are in blue. *p* values under the 0.05, 0.01, and 0.001 thresholds are indicated in italic, in normal font, and in bold, respectively. *x*/*y* reflects the number of genes with a |log2FC| > 2 in the GO category/the total number of genes in the GO category. (**C**) Delta-rank comparison between the responses of non-chimeras and chimeras

After translocation of the *S. pistillata* colonies to 2-m depth, the endosymbiotic algae in the non-chimeric colonies differentially expressed 157 genes (122 over-expressed and 35 under-expressed; Fig. 5A1; Suppl. Table 12) compared to 175 genes in the algae within the chimeras (42 over-expressed and 133 under-expressed; Fig. 5A3; Suppl. Table 13). Only seven DEGs were commonly found in *Symbiodinium* sp. from chimeric and non-chimeric colonies in response to the translocation (Fig. 5A2). In non-chimeric colonies, we identified five significantly (*p* value < 0.05) enriched GO categories (Fig. 5B). The three GO terms over-represented among over-expressed gene were associated to energy production and photosynthesis (e.g., generation of precursor metabolite and energy, photosynthesis). In chimeras, no GO terms were significantly enriched at the 5% error level. Comparison between each enrichment results revealed that the five GO terms enriched in the *Symbiodinium* sp. hosted in non-chimera displayed close delta-rank value to those hosted in chimera which illustrate that only the most significant process responds in the same way (Fig. 5C).

For non-chimeras and in accordance with the results of the enrichment analysis, many genes related to photosynthesis and energy production (i.e., light harvesting complex, oxygen-evolving enhancer, ATP synthase) were differentially expressed. Interestingly, six genes involved in stress response were over-expressed, four cytochrome genes (C1, C6, B5, and B6), one HSP90, and one ferredoxin (Suppl. Table 12). In chimeras, genes involved in photosynthesis were also retrieved over-expressed (e.g., photosystem I reaction center subunit II and oxygen-evolving enhancer protein 1) with only two genes involved in stress response, a HSP70 and a cytochrome peroxidase (Suppl. Table 13).

Similarly to the host, transcriptomic plasticity differences between chimeras and non-chimeras were studied. This was done for the DEGs characterizing the responses of the Symbiodiniaceae hosted in non-chimeras by categorizing the following three patterns: (i) frontloaded genes (i.e., higher basal expression level in chimera), (ii) higher plasticity (i.e., genes with identical or lower basal expression but over- or under-expressed higher in chimeras in response to the translocation), and (iii) frontloaded and higher plasticity (higher basal expression and higher over- or under-expression in chimeras in response to the translocation). As for the host, frontloading was the predominant category (Fig. 4C) and the only one for which significant enrichment was obtained (Fig. 4D). Among the four biological process enriched, three were linked to photosynthesis and energy production (“photosynthesis,” generation of precursor metabolites and energy, and protein-chromophore linkage). At the gene level, gene encoding protein involved in photosynthesis and energy production was retrieved as two genes involved in thermal and light stress response, a flavodoxin and a ferredoxin.

In summary, *Symbiodinium* sp. hosted in non-chimera colonies showed a slight activation of genes and pathways to respond to light and thermal stress. This activation can also be detected in chimeras but it involved very few genes, suggesting a better control of the stress in the holobiont. Interestingly, *Symbiodinium* sp. hosted in chimeras displayed a higher level of expression for genes involved in photosynthetic activity that may results in a higher quantity of energy and energy reserves.

### Chimerism reduces transcriptomic plasticity in *S. pistillata*

**Fig. 6:**
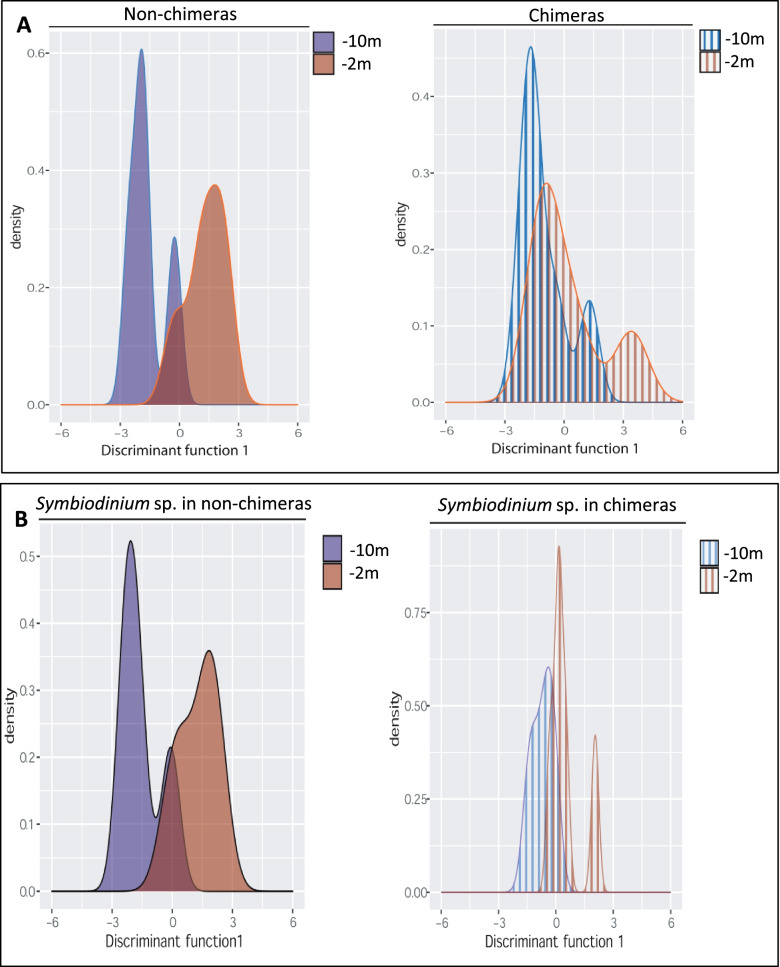
Chimerism reduces transcriptomic plasticity in *S. pistillata.* Discriminant analysis of principal component (DAPC) illustrating the transcriptomic plasticity and the transcriptome diversity (*X* axis) expressed in (**A) ***S. pistillata* and (**B) ***Symbiodinium* sp. hosted in non-chimeras and in chimeras

To assess the degree of transcriptomic plasticity of chimeras compared to non-chimeras, we first conducted a discriminant analysis of principal component (DAPC) based on the transcriptomes of acclimated (i.e., acclimated at 10-m depth) and stressed (i.e., translocated at 2-m depth) non-chimera colonies. We next predicted the coordinates of chimera based on their overall gene expression levels onto the DAPC first discriminant axis. In *S. pistillata* and according to the resulting DAPC, the overall gene expression profiles of the control and stressed chimera showed more overlap than the overall transcriptomic profiles of non-chimera under stress versus control conditions (Fig. 6A).

The DAPC analyses performed from the overall gene expression datasets obtained from the *Symbiodinium* sp. associated with non-chimeric colonies vs. chimeras showed a similar, yet less pronounced, pattern (Fig. 6B). These DAPC analyses illustrate that chimerism reduces transcriptomic plasticity and increases transcriptomic diversity (e.g., more genes expressed over a wider range of levels) at the overall holobiont level but with a much stronger effect in the host.

## Discussion

**Fig. 7:**
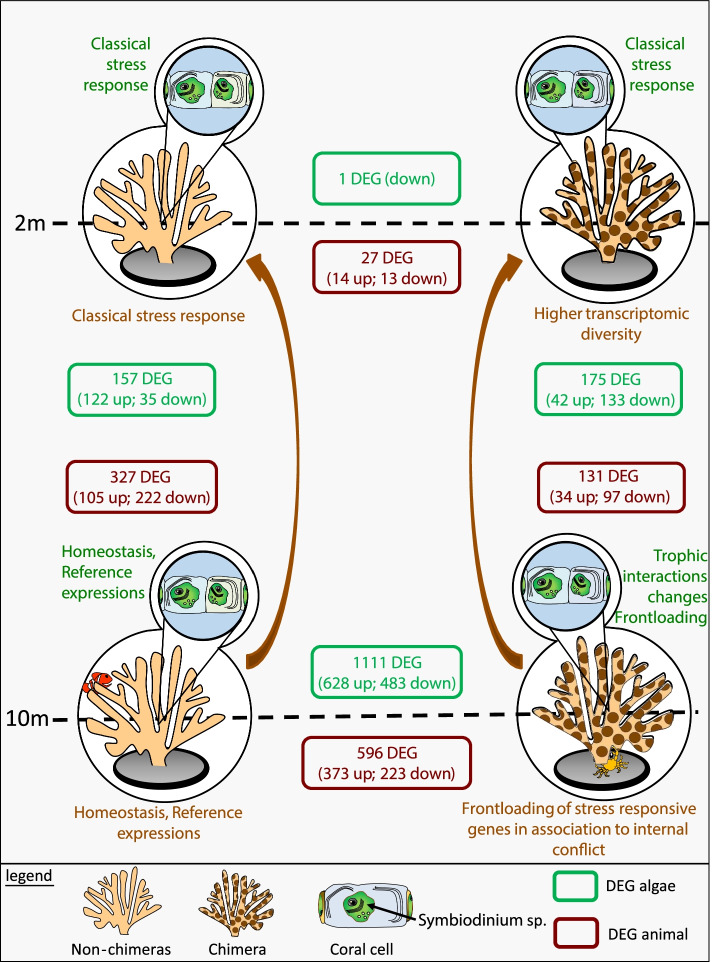
Chimeras are robust entities frontloading stress-responsive genes. Schematic representation of key results and conclusions supporting that chimeras are robust entities characterized by frontloading of stress-responsive genes, lower transcriptomic plasticity, and higher transcriptomic diversity (e.g., more genes expressed over a wider range of levels). When acclimated *in natura* at 10-m depth, non-chimeras (unicolor corals) and chimeras (bi-color corals) differentially express numerous cnidarian (brown box) and algal (green box) genes. In response to an abrupt increase in light intensity and seawater–temperature variability (i.e., translocation to 2-m depth for 48h), the transcriptomic responses of non-chimeric colonies drastically differed from their chimeric counterparts, while at 2-m depth these differences were negligible. The lower transcriptomic plasticity in chimeras, together with the frontloading of stress-responsive genes, illustrates that chimeras are environmental conformers, relying on a strategy of robustness. Brown boxes: cnidarian DEGs; green boxes: *Symbiodinium* sp. DEGs

Here, we show for the first time that chimerism in the coral *Stylophora pistillata* induces transcriptomic modifications that could explain the enhanced tolerance to environmental changes of chimeras *in natura*. We first showed that chimeras and non-chimeras acclimated (for 1 year) at 10-m depth displayed numerous transcriptomic differences both in the cnidarian host and the dinoflagellate endosymbiont (Fig. 7). However, when the corals were subjected to an abrupt environmental change inducing a stress, a translocation from 10- to 2-m depth, gene expression differences between chimeras and non-chimeras became negligible (Fig. 7). Analysis of the transcriptomic responses of chimeric and non-chimeric colonies to the translocation showed that both entities mounted a transcriptomic stress response. In non-chimeric colonies, this response was more potent, but we showed that most of the genes differentially expressed to fight against the stress in non-chimeric colonies were already expressed constitutively at higher levels in the chimeras living under the long-term deep environmental condition (Fig. 4). This phenomenon, called frontloading, has already been shown in corals with better thermotolerance [18, 20]. Thus, chimeras display another interesting trait in comparison to non-chimeric colonies: lower transcriptomic plasticity but wider transcriptomic diversity (i.e., more genes expressed over a wider range of levels; Fig. 5A). The frontloading together with this lower transcriptomic plasticity and wider diversity position chimeras as robust entities better equipped to withstand environmental assaults [26]. This robustness was recently demonstrated in aquaria and *in natura* ecological level, by showing that chimeras display higher survival and thermal tolerance than non-chimeric colonies [27, 28]; these results were also confirmed in our study (Fig. 1B). Finally, it appeared that the phenotype we observed is not associated with a change of the holobiont composition since Symbiodiniaceae assemblages and bacterial microbiota structure were not different between chimeric and non-chimeric colonies.

Coral chimerism is an unexplored biological phenomenon that aggregates ecological and evolutionary concepts associated with these multi-genotype entities [1, 2]. Studies on soft and hard corals, urochordates, and even humans have revealed that chimerism is associated with internal conflicts between the genotypes establishing the chimeras [10, 29, 30]. The existence of these internal conflicts is supported here by the differential expression of many genes related to stress, immune, and tissue reorganization responses [25, 31, 32]. In addition, the over-expression of TEs in chimeric entities suggests that these internal conflicts are perceived up to the genome level, which recall the genomic shock concept developed in other biological models [33].

Taken together, the chronic level of stress established by the internal conflicts between the genotypes of the chimera [10, 29, 30] (this study) and the higher level of phenotypic variation enabled by the expression of multiple genomes within a unique entity [14] (this study) enhance the ability of the chimeras to cope with environmental challenges. Indeed, the results of our study support at the molecular level the notion demonstrated at the ecological level, stating that coral chimerism can buffer the impacts of unpredictable fluctuating environmental conditions [1, 2, 14]. Our experiments show that chimerism promotes transcriptomic changes leading to a shift from a plastic-strategy toward a robust-strategy.

The *Symbiodinium* sp. is surprisingly affected by the coral chimerism. Many genes (*n* = 1111) were differentially expressed between non-chimeras and chimeras despite their acclimation to 10 m over 1 year (Fig. 1). This phenomenon is, however, lowered under stressful conditions (1 DEGs, Fig. 7) probably because the physiological changes induced by the stress are stronger than the one induced by the state of chimerism. Among the genes differentially expressed under long-term regular conditions, many were involved in metabolism, primarily in ion and nutrient transport. Some of these functions have been reported in transcriptomic studies comparing free-living and *in hospite* Symbiodiniaceae [34, 35]. The above suggests that the host’s chimeric status modifies trophic interactions with its symbiont, probably in response to the internal conflicts occurring between the coral genotypes within the chimera [10, 29, 30]. Interestingly, we also showed that *Symbiodinium* sp. responded differently when hosted in chimera and non-chimera. Such differences could be explained by the frontloading of stress and photosynthetic related genes evidenced in chimeras. Frontloading in Symbiodiniaceae is a phenomenon that needs to be better documented but it was recently recorded to be associated coral with higher thermal tolerance thanks to environmental dissipative effects [36]. Alternatively, the difference of responses between the entities, as highlighted in our study, may be attributed to a buffering effect allowed by the higher stress tolerance of the chimeric host. These transcriptomic results on Symbiodiniaceae constitute a novel angle of interpretation on the coral–algal symbiosis, since we obtained opposite results to what is usually concluded; namely that (i) Symbiodiniaceae influence the host transcriptome [37] and (ii) Symbiodiniaceae transcriptomes are relatively stable, even under stressful conditions [38, 39].

## Conclusions

In this study, we showed that chimerism in the stony coral *Stylophora pistillata* is associated with higher survival over a 1-year period and that transcriptomic modifications could explain this robustness gain. A multi-omics approach showed that chimeras lose transcriptomic plasticity and frontload genes responsive to stress which increases chimera ability to face environmental stress. These results put in perspective with the increasing pressure of anthropogenic and global change footprints that threaten the coral reef ecosystems confirm the potential role that coral chimera may play. Indeed, the need for coral reef restoration programs is increasing but many of the strategies developed to rehabilitate a degraded reef are not efficient enough to cope with climate change and anthropogenic threats [3, 40–42], or are still too exploratory to be used or generalized [43]. In this context, coral chimeras have emerged recently [3, 6, 8, 13, 28, 44] as a natural trait that could be harnessed to develop novel active reef restoration practices. Such development would increase the resilience of restored reefs facing a changing world thanks to the higher robustness of coral chimeras [3, 28, 45].

## Methods

### Production and survival of chimeric and non-chimeric colonies

Sexually produced planulae of *Stylophora pistillata* (*Stylophora pistillata* produces larvae only via sexual reproduction) were collected at depth between 2.5 and 4 m along a 300-m stretch of reef in front of the Interuniversity Institute for Marine Sciences (IUI; Eilat) using planula traps [46]. These traps were placed over the same 15 healthy and large *Stylophora pistillata* colonies (15–25 cm in diameter that were spaced by 5–50-m distance) once a week during the 2016, 2018, and 2019 reproductive seasons. The traps were brought to IUI and the larvae content obtained from each mother colony was deposited in a single seawater-filled Petri dish layered with polyester paper (“Maylor paper”; Jolybar, Israel). All dishes were then placed in a water table, partly filled (75% in height) with seawater maintained at ambient temperature. The water in each Petri dish was changed daily. Naturally settled larvae developed into young spat that fused upon direct contact (Fig. 1A1) and formed coral chimeras composed by at least two sibling (same mother colony; unknown sperm donors) individuals [8, 9, 11]. Non-chimeric colonies (single genotype) were produced by avoiding contact. Non-chimeric and chimeric colonies were then carefully removed from the “Maylor paper” (by folding the paper) and glued (super glue; Loctite, Henkel, Germany) to a plastic pin. At the age of 4 months, chimeric and non-chimeric colonies were transferred to a mid-water floating nursery (10-m depth; 29° 32′ 28.92″ N, 34° 58′ 28.62″ E) and were maintained side-by-side [28]. At the age of 12–16 months, the colonies produced in 2016 from seven different mother colonies (mother colony A, B, C, D, E, F, G, Suppl. Table 3) were used for the translocation experiment and molecular analysis (Fig. 1A2). The colonies produced in 2018 and 2019 were maintained under these natural conditions and survival was assessed twice a year until 15 March 2020 when an extreme southern storm in Eilat destroyed the nursery. Due to the removal of chimeras and controls from the nursery by fishermen and recreational diving, the differences in survival between chimeric and non-chimeric corals at 6 and 12 months were assessed at the 95% confidence intervals using the “Cloper – Pearson” method and chi-square test using R software [47].

### Translocation experiment

At the start of the experiment, nine of the 18 chimeras and five of the nine non-chimeras that were still present in the nursery were vertically translocated for 48 h on a floating platform positioned at 2-m depth. To ensure equivalent handling stress, all colonies were handled. The colonies used originated from seven (mothers A, B, C, D, E, F, G) and six (mothers A, B, C, D, E, G) mother colonies, for the chimera and non-chimera set, respectively. Temperature and light were continuously monitored in both platforms using HOBO Pendant Temperature/Light Data Logger®. Coral health was monitored daily with the coral reef watch color chart [48]. Chimeric and non-chimeric colonies from both platforms were collected (using SCUBA) and immediately put in RNAlater solution, stored at 4°C for 24h and then at −20°C until RNA and DNA extractions.

### RNA and DNA extraction

Each colony was separately grounded in liquid nitrogen in 50-ml stainless steel bowls with 20-mm-diameter grinding balls (Retsch MM400 mill). RNA extraction was achieved from 200 mg of powdered corals mixed with 1 ml of Trizol reagent (Invitrogen). After precipitation with the high salt solution protocol, RNA pellet was resuspended in 40 μl of RNAsecure reagent (Ambion) and RNase were heat-inactivated by an incubation at 65°C for 10 min. RNA concentration and purity were checked using a Nanodrop ND-1000 spectrometer (Thermo Scientific). The appropriate amount of RNA was next treated with the TURBO DNase kit (Ambion). Total RNAs was cleaned using the RNeasy Power Clean Pro Cleanup kit (Qiagen). RNA integrity and quantification were analyzed by capillary electrophoresis on a BioAnalyzer 2100 (Agilent). DNA was extracted from an aliquot of the powdered corals using a DNAeasy Blood and Tissue kit (Qiagen) and was quantified by spectrophotometry (NanoDrop). All protocols followed the manufacturers’ instructions.

### Validation of chimeric status

Six highly variable microsatellite loci were used to validate the chimeric status of each *Stylophora pistillata* colony following a published protocol [49] with some modifications. Each reaction contained 1 μl of mixed genomic DNA, 0.1μl of primer set (10μM), and 5 μl of 2XTaq PCR Master MIX (Tiangen Biotech, Beijing) in 10 μl final volume. Amplification conditions were 94°C for 5 min, 25 cycles of 30 s at 94°C, 90 s at 57°C, and 60 s at 72°C, followed by 30 min at 60°C. For each sample, 1μl of the PCR product was added to 8.6 μl of formamide and 0.4μl of LIZ size marker (MapMarkerDY632 50–500bp) and was analyzed at Rapport Medical School, Israel, using 3500xl genetic analyzer (Life Technology). Analyses were performed using GeneMapper software (Life Technology) and Thermo Fisher Cloud Microsatellite Analysis Software (https://apps.thermofisher.com/apps/spa/#/dashboard). As some chimeras were siblings (same mother colony; unknown sperm donors), a chimeric state was identified with at least one locus presenting more than two alleles.

This validation was further confirmed by variant calling analysis performed with the RNA-seq data. For this purpose, raw reads were filtered according to their quality (Phred score >30) and adaptors were removed using Trim Galore! [50]. Cleaned read pairs were mapped (default parameters) on the reference genome of *Stylophora pistillata* [23] with RNA-STAR [51]. Mapped paired reads were then filtered with samtools [52]: (i) properly paired reads were selected; (ii) paired reads mapped outside of genes were removed; (iii) 8 million mapped paired reads were randomly subsampled to apply the same sequencing effort to each sample. Variant calling was performed sample by sample with freebayes [53] using parameters dedicated to call variants from pooled sequencing data (--pooled-continuous) and the number of expected genomes per sample was set accordingly to the number of genotypes used to create the chimera (-p option) [52]. To maximize the chance of detecting chimeric entities, multiple nucleotide polymorphisms (MNPs, e.g., short haplotype containing several SNPs) were extracted from the vcf file with bcftools view function and general statistics on these lists of MNPs were computed with bcftools stats function. Scripts for bioinformatics and bioanalysis used in this study are available in Additional file 3.

### Dual transcriptome analysis by RNA-seq

RNA-seq library construction and sequencing: Directional cDNA libraries were prepared using the PolyA+ Stranded SENSE mRNA Sample Preparation kit (Lexogen) according to the manufacturer’s instructions. Libraries were then sequenced on an Illumina NextSeq550 instrument in paired-end reads of 2 × 150 bp at the Bio-Environment platform (University of Perpignan, France). Sequencing data are available for download on sextant 10.12770/86ff6c42-3771-45eb-9164-a18159d7c7fe [54] and on SRA bioproject PRJNA858201 [55].

Bioinformatics analyses were performed under a local galaxy instance (https://bioinfo.univ-perp.fr/). Phred scores were checked using FastQC [56] and reads were filtered according to their quality using Trim Galore! [50]: The 10 first bases from the 5′ end and the last bases at the 3′ end were trimmed, adaptors were removed, and reads with Phred score <20 were discarded. For dual RNA-seq analyses, cleaned reads were mapped to the *S. pistillata* [23] and *Symbiodinium microadriaticum* [24] reference genomes using RNA-STAR [51] using default parameters. HTSeq-count [57] was used to count the number of reads overlapping annotated genes (mode union, minimum alignment quality of 10). Differential gene expression level analyses were done with the mother colony of interest as a covariate (design = ~ treatment + mother_colony) and normalized log-transformed counts for plasticity analysis were transformed to control the mother-colony effect (function limma::removeBatchEffect) [58]. Significant fold changes between treatments were considered for adjusted *p* value on multiple testing using the Benjamini–Hochberg correction (false discovery rate FDR < 0.05). Over/under-expressed biological functions were identified by a gene ontology (GO) analysis using adaptive clustering in association to either a rank-based statistical test (Mann–Whitney *U* test; continuous value = log2 fold change) or a Fisher exact test (binary value; 0/1) as implemented in the R script GO_MWU (https://github.com/z0on/GO_MWU). Clustering parameters were largest = 0.1; smallest = 10; clusterCutHeight = 0.5. A gene ontology category was considered significantly enriched under a 5% FDR threshold. Delta-rank comparison between enrichment results were performed with GO-Delta-Rank-Correlation script with R [59].

Transcriptomic plasticities were analyzed using a discriminant analysis of principal components (DAPC [19, 60];), based on the matrices of transcript abundance. Specifically, we first ran a DAPC analysis considering the non-chimera samples exposed at 10-m and 2-m depth as predefined groups using the “Adegenet” package implemented in R. This allowed us to illustrate the transcriptomic shift in non-chimera due to the translocation. We next predicted the coordinates of the chimera exposed to the 10-m and 2-m depth treatment into the unique discriminant function of the initial DAPC.

Scripts for bioinformatics and bioanalysis used in this study are available in Additional file 3.

### Microbial community analyses

#### Amplicon sequencing

A bacterial 16S rDNA amplicon library was generated for each sample, using the 341F (CCTACGGGNGGCWGCAG) and 805R (GACTACHVGGGTATCTAATCC) primers, which target the variable V3/V4 loops [61]. The Symbiodiniaceae ITS2 amplicon libraries were generated with specific primers targeting ca. 350-bp sequence (ITS2-F GTGAATTGCAGAACTCCGTG; ITS2-R CCTCCGCTTACTTATATGCTT) [62, 63]. For both markers, paired-end sequencing using a 2 × 250 bp read length was performed on a MiSeq instrument (Illumina) at the Bio-Environment platform (University of Perpignan, France) using the v2 chemistry, according to the manufacturer’s protocol. Sequencing data are available for download on sextant 10.12770/86ff6c42-3771-45eb-9164-a18159d7c7fe and on SRA bioproject PRJNA858201 [55].

The FROGS pipeline implemented on a Galaxy platform (http://sigenae-workbench.toulouse.inra.fr/galaxy/) was used for data processing [64]. In brief, paired reads were merged using FLASH [65] and after removal of primer/adapters using cutadapt [66], de novo clustering was performed with SWARM [67], using a local clustering threshold with an aggregation distance of 3. Chimeric sequences were removed using VSEARCH [68]. The dataset was checked for singletons, and affiliations for 16S amplicons [69] were performed using Blast+ against the Silva SSU database (release 132, December 2017). The *Symbiodiniaceae* clade was assessed using Blastn against GenBank. An OTU table in standard BIOM format with taxonomic affiliation was produced for subsequent analyses. For community composition analysis, we used the *phyloseq* R package [70] to infer alpha diversity metrics at the OTU level and beta diversity (between sample similarity) from the OTU table. Community similarity was assessed by principal coordinate analysis using the Bray–Curtis distance matrices. Corrections based on multiple testing were performed using FDR [71]. For all analyses, the threshold significance level was set at 5%.

Scripts for bioinformatics and bioanalysis used in this study are available in Additional file 3.

## Supplementary Information

**Additional file 1: Supplementary table 1**: Temperature and light intensity data set for the shallow (2m) and deep (10m) platforms during the 48h of experiment. **Supplementary table 2**: Temperatures and light regimes at the sallow (2m) and deep (10m) platforms during the 48h of experiment. **Supplementary table 3**: Metadata associated to each sample used in the study; treatment, chimeric statue, expected number of hots genome, sample ID, mother colony of origin, number of tri-allelic (or more) locus, microsatellite dysplaying tri-allelic (or more) locus, number of MNPs, number of MNPs with n allele>2, Relative quantity of MNPs with n allele>2. **Supplementary table 4**: ITS2 OTU table. **Supplementary table 5**: 16S OTU table. **Supplementary table 6**: Mapping results against the host or symbiont genome. **Supplementary table 7**: Differentially expressed genes between non-chimeras host and chimeras host at 10m. **Supplementary table 8**: Differentially expressed genes between non-chimeras host and chimeras host at 2m. **Supplementary table 9**: Differentially expressed genes between symbiont hosted in non-chimeras and in chimeras at 10m. **Supplementary table 10**: Differentially expressed genes between symbiont hosted in non-chimeras and in chimeras at 2m. **Supplementary table 11**: Differentially expressed genes between 2m and 10m in non-chimeras. **Supplementary table 12**: Differentially expressed genes between 2m and 10m in chimeras. **Supplementary table 13**: Differentially expressed genes between 2m and 10m in symbiont hosted in non-chimeras. **Supplementary table 12:** Differentially expressed genes between 2m and 10m in symbiont hosted in chimeras.

**Additional file 2: Supplementary figure 1**: Bacterial microbiomes associated with non-chimeric colonies and chimeras at 10 and 2m depth. (A) Alpha diversity index: (A1) observed; (A2) Chao1; (A3) Shannon. Significant differences (MANOVA) are marked by an asterisk (*). (B) PCoA Bray-Curtis dissimilarity index (beta diversity). (C) Bacterial community composition at the Family level. Red=Chimeras deep platform; Green=Chimeras shallow platform; Blue= Controls deep platform; Purple=Controls shallow platform. **Supplementary figure 2**: Hierarchical clustering performed using DEseq2 rlog normalized RNA-seq data. Hierarchical clustering of the 20 transcriptomes of *Stylophora pistillata* (A) and *Symbiodinium microadriaticum* (B) samples. Each sample is named by its chimeric state (Chimera/Non-chimera), treatment (-2m/-10m) and mother colony of origin (A, B, C, D, E, F, G). **Supplementary figure 3**: Principal component analysis using DEseq2 rlog normalized RNA-seq data. PCA of the 20 transcriptomes of *Stylophora pistillata* (A) and *Symbiodinium microadriaticum* (B) samples.

**Additional file 3.** Scripts used for bioinformatics and biostatistics.

## Data Availability

Sequencing data are publicly available on sextant 10.12770/86ff6c42-3771-45eb-9164-a18159d7c7fe and on SRA bioproject PRJNA858201.

## Abbreviations

C3: Complement factor 3
C1q: Complement factor 1q
DAPC: Discriminant analysis of principal component
DEG: Differentially expressed gene
GO: Gene Ontology
HSP: Heat shock protein
HSF: Heat shock factor
IRF: Interferon regulatory factor
log2FD: log2 fold change
MNP: Multiple nucleotide polymorphism
MYD88: Myeloid differentiation primary response 88
PCA: Principal component analysis
OUT: Operational taxonomic unit
ROS: Reactive oxygen species
SIG: Stimulator of interferon gene
*S. microadriaticum*: *Symbiodinium microadriaticum*
*S. pistillata*: *Stylophora pistillata*
TE: Transposable element
TRAF: TNF receptor-associated factors
