# Supplementary figures and images for: Frontloading of stress response genes enhances robustness to environmental change in chimeric corals
Source: BMC Biol. 2022 Jul 26;20:167. doi: 10.1186/s12915-022-01371-7 (PMC9316358; doi:10.1186/s12915-022-01371-7)

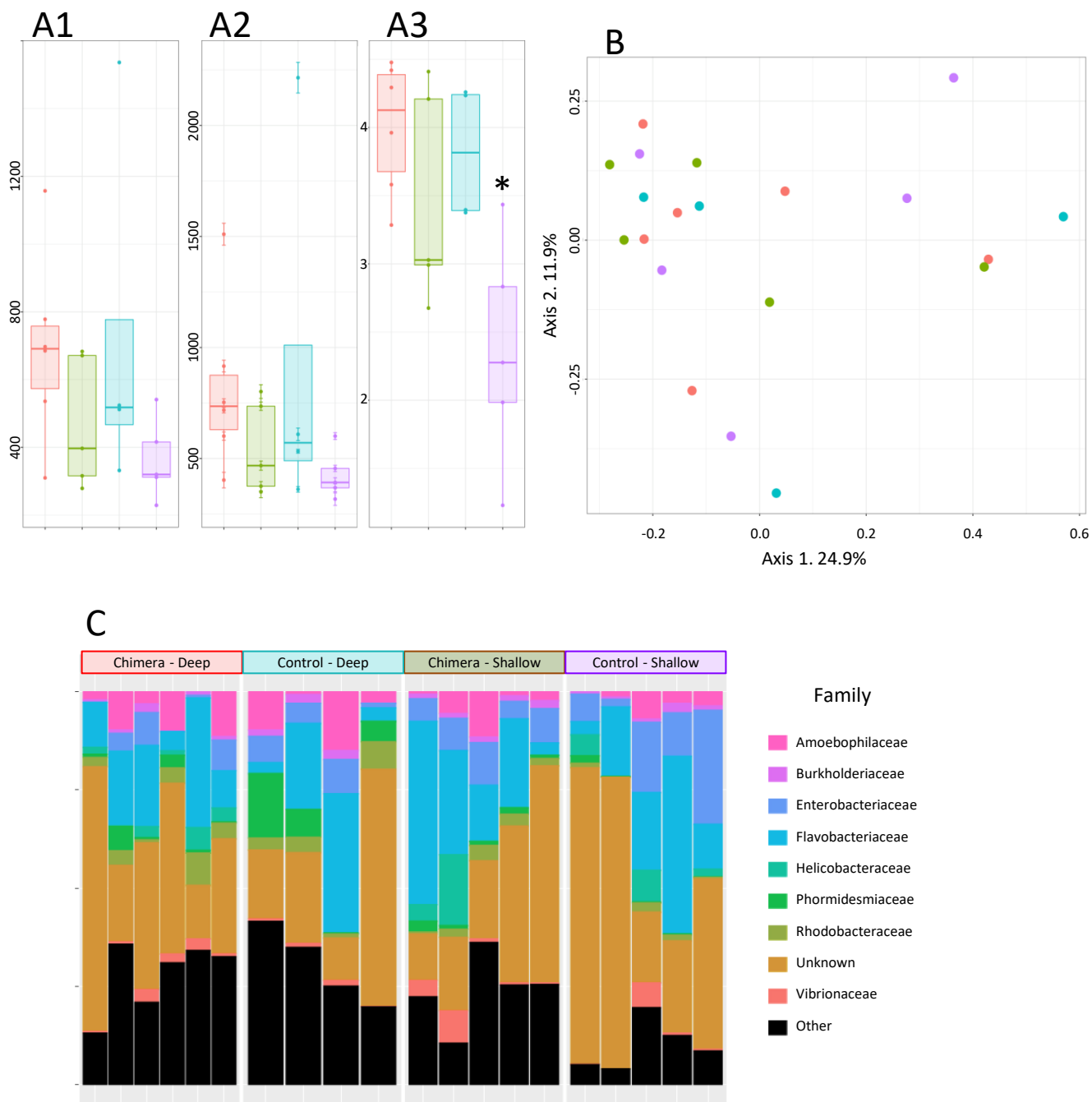

Figure S1

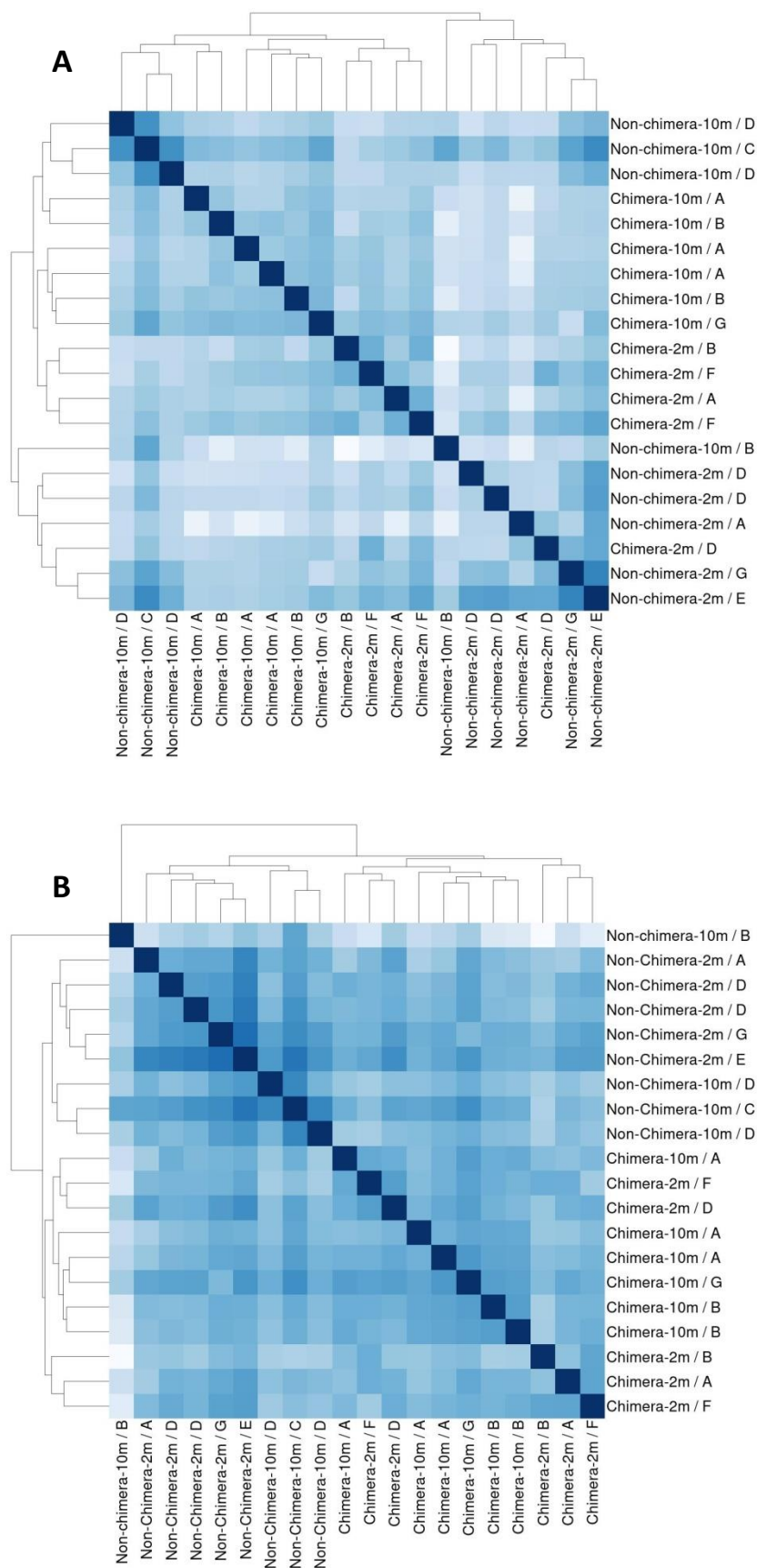

Figure S2

**A**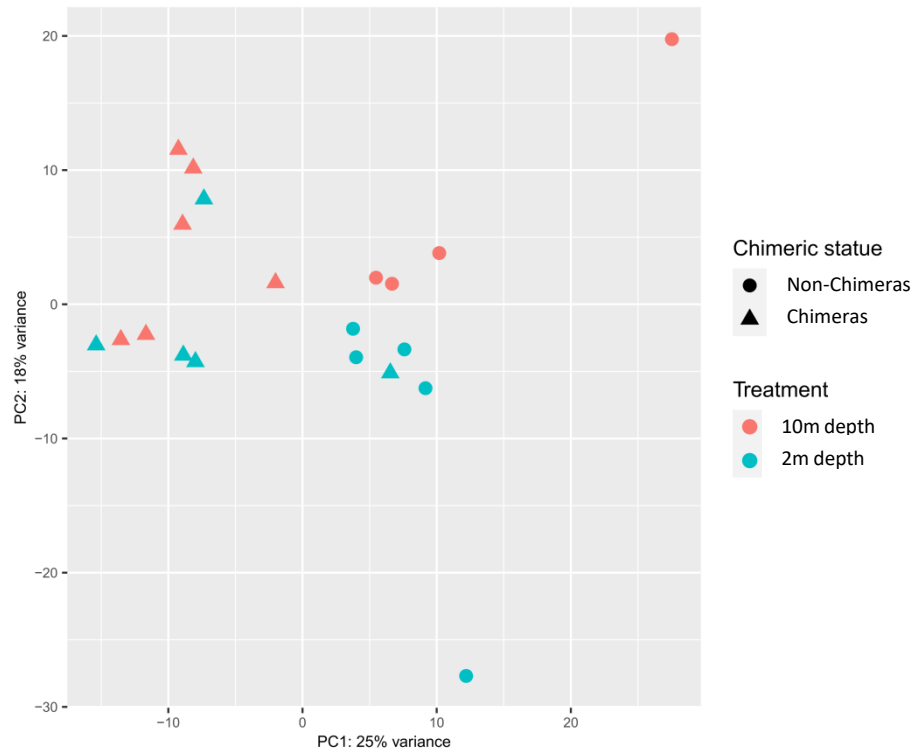**B**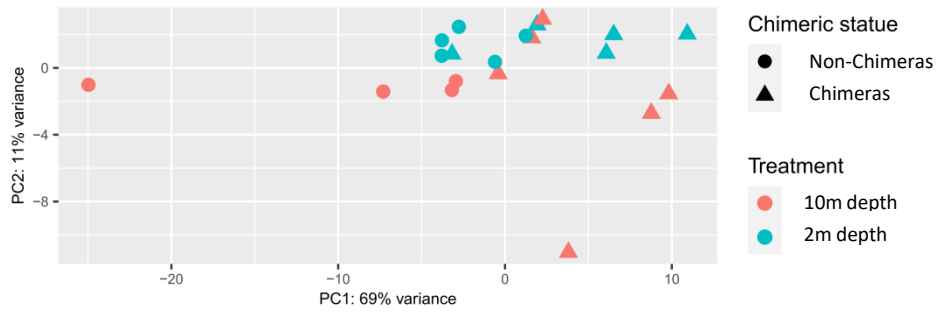

Figure S3

Supplement: Supplementary file 2 — Additional file 2: Supplementary figure 1: Bacterial microbiomes associated with non-chimeric colonies and chimeras at 10 and 2m depth. (A) Alpha diversity index: (A1) observed; (A2) Chao1; (A3) Shannon. Significant differences (MANOVA) are marked by an asterisk (*). (B) PCoA Bray-Curtis dissimilarity index (beta diversity). (C) Bacterial community composition at the Family level. Red=Chimeras deep platform; Green=Chimeras shallow platform; Blue= Controls deep platform; Purple=Controls shallow platform. Supplementary figure 2: Hierarchical clustering performed using DEseq2 rlog normalized RNA-seq data. Hierarchical clustering of the 20 transcriptomes of Stylophora pistillata (A) and Symbiodinium microadriaticum (B) samples. Each sample is named by its chimeric state (Chimera/Non-chimera), treatment (-2m/-10m) and mother colony of origin (A, B, C, D, E, F, G). Supplementary figure 3: Principal component analysis using DEseq2 rlog normalized RNA-seq data. PCA of the 20 transcriptomes of Stylophora pistillata (A) and Symbiodinium microadriaticum (B) samples. [file 12915_2022_1371_MOESM2_ESM.pdf]
