# Supplementary material for: Frontloading of stress response genes enhances robustness to environmental change in chimeric corals
Source: BMC Biol. 2022 Jul 26;20:167. doi: 10.1186/s12915-022-01371-7 (PMC9316358; doi:10.1186/s12915-022-01371-7)
Supplement: Supplementary file 3 — Additional file 3. Scripts used for bioinformatics and biostatistics. [file 12915_2022_1371_MOESM3_ESM.pdf]

Bioinformatic and biostatistic scripts :

Galaxy Script for FastQC

Content homepage: http://www.bioinformatics.babraham.ac.uk/projects/fastqc/
Development repository: https://github.com/galaxyproject/tools-iuc/tree/master/tools/fastqc
Link to this repository revision: https://toolshed.g2.bx.psu.edu/view/devteam/fastqc/ff9530579d1f

fastqc --outdir '/home1/’ --quiet --extract -f 'fastq' 'input.gz' fastqc_data.txt output.txt /*\.html output.html

Galaxy Script for Trim Galore!

Content homepage: http://www.bioinformatics.babraham.ac.uk/projects/trim_galore/
Development repository: https://github.com/bgruening/galaxytools/tree/master/tools/trim_galore
Link to this repository: https://toolshed.g2.bx.psu.edu/view/bgruening/trim_galore/cd7e644cae1d

trim_galore --phred33   --quality 28 --stringency 1 -e 0.1 --length 30   --clip_R2 9   --output_dir ./   --illumina --three_prime_clip_R1 1 --paired   --three_prime_clip_R2 1 input_1.fastq.gz input_2.fastq.gz

Galaxy Script for RNAstar

Content homepage: https://github.com/alexdobin/STAR
Development repository: https://github.com/galaxyproject/tools-iuc/tree/master/tools/rgrnastar
Link to this repository revision: https://toolshed.g2.bx.psu.edu/view/iuc/rgrnastar/99b17b74a8cd

STAR --runMode genomeGenerate --genomeDir 'tempstargenomedir' --genomeFastaFiles --runThreadN ${GALAXY_SLOTS:-4} && STAR --runThreadN ${GALAXY_SLOTS:-4} --genomeLoad NoSharedMemory --genomeDir   --readFilesIn --readFilesCommand zcat   --outSAMtype BAM SortedByCoordinate   --outSAMattributes Standard --outSAMstrandField None --outFilterIntronMotifs None --outFilterIntronStrands RemoveInconsistentStrands --outSAMunmapped None --outSAMprimaryFlag OneBestScore --outSAMmapqUnique "255" --outFilterType Normal --outFilterMultimapScoreRange "1" --outFilterMultimapNmax "10" --outFilterMismatchNmax "10" --outFilterMismatchNoverLmax "0.3" --outFilterMismatchNoverReadLmax "1.0" --outFilterScoreMin "0" --outFilterScoreMinOverLread "0.66" --outFilterMatchNmin "0" --outFilterMatchNminOverLread "0.66" --outSAMmultNmax "-1" --outSAMtlen "1" --outBAMsortingBinsN "50"   --seedSearchStartLmax "50" --seedSearchStartLmaxOverLread "1.0" --seedSearchLmax "0" --seedMultimapNmax "10000" --seedPerReadNmax "1000" --seedPerWindowNmax "50" --seedNoneLociPerWindow "10" --alignIntronMin "21" --alignIntronMax "0" --alignMatesGapMax "0" --alignSJoverhangMin "5" --alignSJDBoverhangMin "3" --alignSplicedMateMapLmin "0" --alignSplicedMateMapLminOverLmate "0.66" --alignWindowsPerReadNmax "10000" --alignTranscriptsPerWindowNmax "100" --alignTranscriptsPerReadNmax "10000" --alignEndsType

```
Local  --twopassMode "None"   --limitBAMsortRAM "0" --limitOutSJoneRead "1000" --
limitOutSJcollapsed "1000000" --limitSjdbInsertNsj "1000000"
```

Galaxy Script for HTseq_Count:

Content homepage: https://readthedocs.org/projects/htseq/
Development repository: https://github.com/galaxyproject/tools-iuc/tree/master/tools/htseq_count
Link to this repository revision: https://toolshed.g2.bx.psu.edu/view/lparsons/htseq_count/620d5603d1a8

```
htseq-count --mode=union --stranded=yes --minaqual=10 --type='gene' --idattr='Name'   --
order=name --format=sam 'name_sorted_alignment.sam'
```

Galaxy Script for samtools view

Content homepage: https://www.htslib.org/
Development repository: viewhttps://github.com/galaxyproject/tools-iuc/tree/master/tool_collections/samtools/samtools_view
Link to this repository revision: https://toolshed.g2.bx.psu.edu/view/iuc/samtools_merge/740ce0a18f0d

```
addthreads=${GALAXY_SLOTS:-1} && (( addthreads-- )) &&     ln -s '/home/datawork-bioinfo-
ss/galaxy/galaxy-prod/files/000/242/dataset_242466.dat' infile && ln -s '/home/datawork-bioinfo-
ss/galaxy/galaxy-prod/files/_metadata_files/002/metadata_2624.dat' infile.bai &&
sample_fragment=`samtools idxstats infile | awk '{s+=$4+$3} END {frac=8000000/s; print(frac < 1 ? "-
s " 10+frac : "")}'` && samtools view -@ $addthreads  -b  "${sample_fragment}"  -h  -o outfile    infile
```

Galaxy_script for metabarcoding

Script for Freebayes

```
freebayes -f Spis_genome_scaffold_final.fa -p 2 --pooled-continuous filtered_alignments.bam  >
var.vcf
```

R script for DEseq2 analysis https://bioconductor.org/packages/release/bioc/html/DESeq2.html

```
library(tidyverse)

title_suffix1 <- "deep vs shallow control"

title_suffix2 <- "deep vs shallow chimeric"

title_suffix3 <- "chimera vs control at shallow"
```

```
title_suffix4 <- "chimera vs control at deep"

#remanded treshold1 = 1, remanded treshold2 = 10, Deseq2 treshold1 seems to be = 0, Deseq2 treshold2 seems to be = 0

treshold1 = 0

treshold2 = 0

#load count table

#list files useful for the loop

htseq_tables <- list.files(here::here("data", "corail"), full.names = TRUE)

#prepare the vector

count_data <- NULL

#do a loop to load and merge all tables grom htseq count

for (i in htseq_tables) {

 #load the tables from htseq count

 dat <- readr::read_delim(i, col_names = FALSE, delim = "\t")

 #remove all the characters before the last "/"

 i <- sub(".*\\/","", i)

 #remove the last part of the text

 i <- sub("_htseq-count_Stylophora].tabular", "", i)

 #rename the columns of the dataframe

 colnames(dat) <- c(paste("gene", i, sep = "_"), paste("count", i, sep = "_"))

 #bind columns

 count_data <- dplyr::bind_cols(count_data, dat)

}

#load metadata

metadata <- readr::read_csv(here::here("data/metadata.csv"), col_names = TRUE)

#for an incomprehensible reason two additional columns were created, we remove them

count_data <- count_data[,-c(35,36)]
```

```
#to use DESeq2::DESeqDataSetFromMatrix the row names of the dataframe have to correspond to the genes and remove other genes colums

count_data <- count_data %>%

  remove_rownames %>%

  column_to_rownames(var="gene_Deep_Ch_102") %>%

  select(-contains("gene"))

#rename columns name to suitable names

colnames(metadata)[colnames(metadata)== "Chimeric statue"] <- "chimeric_statue"

colnames(metadata)[colnames(metadata)== "Mother colony of origin"] <- "mother_colony"

#to create a DESeqDataSet object "Rows of colData (here metadata object) correspond to columns of countData (count_data object)"

#rename columns of the count_data

colnames(count_data) <- sub(".*\\_", "", colnames(count_data))

#change row names of metadata to correspond to the sample names

metadata <- metadata %>% remove_rownames %>% column_to_rownames(var="sample_ID") %>% select(-"Number of expected genome")

#reorder the dataframe in order the firt row of metadata corresond to the first column of count_data

metadata <- metadata[order(match(rownames(metadata), colnames(count_data))),]

#transform values of the metadata as factor

metadata$Treatment <- as.factor(metadata$Treatment)

metadata$chimeric_statue <- as.factor(metadata$chimeric_statue)

metadata$mother_colony <- as.factor(metadata$mother_colony)

metadata_shallow <- metadata[metadata$Treatment %in% "Shallow",]

metadata_shallow <- metadata_shallow[,c("chimeric_statue", "mother_colony")]

metadata_shallow$chimeric_statue <- factor(metadata_shallow$chimeric_statue, levels = c("Control","Chimera"))

count_data_shallow <- count_data[,rownames(metadata_shallow)]

ddsMat <- DESeq2::DESeqDataSetFromMatrix(countData = count_data_shallow,

                                colData = metadata_shallow,
```

```
                                  design = ~ chimeric_statue + mother_colony)
keep <- rowSums(DESeq2::counts(ddsMat)) > treshold1
ddsMat <- ddsMat[keep,]
rld <- DESeq2::rlog(ddsMat, blind = FALSE)
SummarizedExperiment::assay(rld) <- limma::removeBatchEffect(SummarizedExperiment::assay(rld),
                        batch = SummarizedExperiment::colData(ddsMat)[,"mother_colony"],
                        design = model.matrix(~chimeric_statue, SummarizedExperiment::colData(rld)))
#Plot distance matrix
sampleDists <- dist(t(SummarizedExperiment::assay(rld)))
sampleDistMatrix <- as.matrix(sampleDists)
rownames(sampleDistMatrix) <- metadata_shallow$chimeric_statue
colnames(sampleDistMatrix) <- metadata_shallow$chimeric_statue
library("pheatmap")
library("RColorBrewer")
colors <- colorRampPalette(rev(brewer.pal(9, "Blues")))(255)
pheatmap(sampleDistMatrix,
     clustering_distance_rows = sampleDists,
     clustering_distance_cols = sampleDists,
     col = colors)

#plot PCA
pcaData <- DESeq2::plotPCA(rld, intgroup = "chimeric_statue", returnData = TRUE)
percentVar <- round(100 * attr(pcaData, "percentVar"))
library(ggplot2)
ggplot(pcaData, aes(x = PC1, y = PC2, color = chimeric_statue)) +
  geom_point(size =3) +
  xlab(paste0("PC1: ", percentVar[1], "% variance")) +
```

```
  ylab(paste0("PC2: ", percentVar[2], "% variance")) +

  coord_fixed() +

  ggtitle("PCA with rlog data: control vs chimera at shallow")


#DE analysis

ddsMat <- DESeq2::estimateSizeFactors(ddsMat)

# nc <-  DESeq2::counts(ddsMat, normalized=TRUE)

# filter <- rowSums(nc >= 10) >= 2

# ddsMat <- ddsMat[filter,]

keep <- rowSums(DESeq2::counts(ddsMat)) >= treshold2

ddsMat <- ddsMat[keep,]

ddsMat <- DESeq2::estimateDispersions(ddsMat)

deseq <- DESeq2::nbinomWaldTest(ddsMat, maxit=500)

result_table_chimeraVScontrol_shallow <- DESeq2::results(deseq,

                                contrast=c("chimeric_statue", "Chimera", "Control"))

#write.csv(as.data.frame(result_table_chimeraVScontrol_shallow),
here::here("Other/results_en_cours/result_table_chimeraVScontrol_shallow.csv"))

head(as.data.frame(result_table_chimeraVScontrol_shallow))

#Plot MAplot

library(apeglm)

DESeq2::resultsNames(deseq)

DESeq2::plotMA(result_table_chimeraVScontrol_shallow,

            main= paste("MA-plot for", title_suffix1),

            ylim=range(result_table_chimeraVScontrol_shallow$log2FoldChange, na.rm=TRUE))

#Plot Histogram of p-value

hist(result_table_chimeraVScontrol_shallow$pvalue[result_table_chimeraVScontrol_shallow$baseM
ean > 1], breaks = 0:20/20, col = "grey50", border = "white", main=paste("Histogram of p-values for",
title_suffix1))
```

R script for GO_MWU analysis https://github.com/z0on/GO_MWU

input="non_chimeraVSchimera_10m.csv" # two columns of comma-separated values: gene id, log2FD if FDR<0.05; 0 if FDR>0.05 OR gene id, 0 or 1 for list comparison

goAnnotations="Transcriptome_Stylophora_GOannot2.tab.txt" # two-column, tab-delimited, one line per gene, multiple GO terms separated by semicolon.

goDatabase="go.obo" # download from http://www.geneontology.org/GO.downloads.ontology.shtml

goDivision="BP"

source("gomwu.functions.R")

Calculating stats. gomwuStats(input, goDatabase, goAnnotations, goDivision,

perlPath="perl"

largest=0.1, # a GO category will not be considered if it contains more than 10% the total number of genes

smallest=10, # a GO category should contain at least 10 genes to be considered

clusterCutHeight=0.5, # 50% threshold for merging similar (gene-sharing) terms.

Plotting results

ape()

gomwuPlot(input,goAnnotations,goDivision,

absValue=2, # genes with the measure value exceeding this will be counted as "good genes" and correspond to gene with FDR<0.05 or gene present in the test list for binary classification

level1=0.05, # FDR threshold for plotting, italic

level2=0.01, # FDR cutoff to print in regular (not italic) font.

level3=0.001, # FDR cutoff to print in large bold font.

txtsize=1.8,

treeHeight=0.6)

R script for GO_Dekta-Rank_Correlation https://github.com/z0on/GO_MWU

#setwd

data1=read.table("MWU_BP_Res_chim_FD.csv",header=T)

```
data2=read.table("MWU_BP_Res_cont_FD.csv",header=T)

goods=intersect(data1$term,data2$term)
goods=unique(as.character(c(data1$term[data1$p.adj<=1],data2$term[data2$p.adj<=1])))
length(goods)

data1=data1[data1$term %in% goods,]
data2=data2[data2$term %in% goods,]

# all overlapping GO terms
ress=merge(data1,data2,by="term")
plot(delta.rank.x~delta.rank.y,ress,xlab="Chimera", ylab="Control",mgp=c(2.3,1,0))
abline(v=0,lty=3)
abline(h=0,lty=3)

# GO terms highly significant in any of the two datasets
sigs=(ress$p.adj.x<=0.05 | ress$p.adj.y<=0.05)
sum(sigs) # 71
plot(delta.rank.x~delta.rank.y,ress[sigs,],xlab="Chimera", ylab="Control",mgp=c(2.3,1,0))
abline(v=0,lty=3)
abline(h=0,lty=3)

# GO terms signifcant in both datasets
sigs=(ress$p.adj.x<=0.05 & ress$p.adj.y<=0.05)
sum(sigs) # 20
plot(delta.rank.x~delta.rank.y,ress[sigs,],xlab="Chimera", ylab="Control",mgp=c(2.3,1,0))
abline(v=0,lty=3)
```

```
abline(h=0,lty=3)
```
